# Effect of exopolysaccharides from cariogenic bacteria on human gingival fibroblasts

**DOI:** 10.7150/ijms.57221

**Published:** 2021-05-13

**Authors:** Anna K. Szkaradkiewicz-Karpińska, Andrzej Szkaradkiewicz

**Affiliations:** 1 Department of Preclinical Conservative Dentistry and Preclinical Endodontics, University of Medical Sciences, 60-812 Poznań, Poland.; 2 Institute of Health and Physical Culture, State Higher Vocational School, 64-100 Leszno, Poland.

**Keywords:** dental caries, *Streptococcus mutans*, * Lactobacillus acidophilus*, exopolysaccharides, ATP, gingivitis

## Abstract

Bacterial biofilm (dental plaque) plays a key role in caries etiopathogenesis and chronic periodontitis in humans. Dental plaque formation is determined by exopolysaccharides (EPSs) produced by cariogenic and periopathogenic bacteria. The most frequent cariogenic bacteria include oral streptococci (in particular *S. mutans*) and lactobacilli (most frequently *L. acidophilus*). In turn, the dominant periopathogen in periodontitis is *Porphyromonas gingivalis.* Development of dental caries is often accompanied with gingivitis constituting the mildest form of periodontal disease. Basic cellular components of the gingiva tissue are fibroblasts the damage of which determines the progression of chronic periodontitis. Due to insufficient knowledge of the direct effect of dental plaque on metabolic activity of the fibroblasts, this work analyses the effect of EPSs produced by *S. mutans* and *L. acidophilus* strains (H_2_O_2_-producing and H_2_O_2_-not producing) on ATP levels in human gingival fibroblasts (HGF-1) and their viability. EPSs produced in 48-hours bacterial cultures were isolated by precipitation method and quantitatively determined by phenol - sulphuric acid assay. ATP levels in HGF-1 were evaluated using a luminescence test, and cell viability was estimated using fluorescence test. The tests have proven that EPS from *S. mutans* did not affect the levels of ATP in HGF-1. Whereas EPS derived from *L. acidophilus* strains, irrespective of the tested strain, significantly increased ATP levels in HGF-1. The analysed EPSs did not affect the viability of cells. The tests presented in this work show that EPSs from cariogenic bacteria have no cytotoxic effect on HGF-1. At the same time, the results provide new data indicating that EPSs from selected oral lactobacilli may have stimulating effect on the synthesis of ATP in gingival fibroblasts which increases their energetic potential and takes a protective effect.

## Introduction

It is already known that the so called cariogenic bacteria, mainly oral streptococci, especially *Streptococcus mutans* and lactobacilli, most frequently *Lactobacillus acidophilus*, played a key role in etiopathogenesis in dental caries 1, 2, 3. These bacteria demonstrate intensive acidogenicity and acid tolerance 2, 4. Moreover, they have the ability to produce extracellular polysaccharides (EPS) which allow accumulation of bacteria on the surface of tooth enamel and creation of dental plaque (cariogenic biofilm), in the consequence of which tooth decay develops 5, 6, 7, 8. On the other hand, dental plaque accumulation on teeth adjacent to the gingiva induces gingivitis, which constitutes the mildest form of periodontal disease and affects 50-90% of adults worldwide 9. Dental plaque associated with periodontal disease is built of anaerobic bacteria (the so called periopathogens), and the dominant periopathogen is *Porphyromonas gingivalis* 10, 11. Previous studies have proven that extracellular products of selective periopathogens reduce the synthesis of adenosine triphosphate (ATP) in gingival fibroblasts 12. Contrary to those results, it was determined that extracellular products of selected *Lactobacillus acidophilus* strains cultures may stimulate ATP synthesis in gingival fibroblasts 12. However, the tests performed concerned only supernatants obtained from a 24-hours bacteria culture. Considering the above data, the purpose of this work was to analyse the effect of EPSs produced by *S. mutans* and *L. acidophilus* on ATP levels in human gingival fibroblasts.

## Materials and Methods

### Bacterial strains and culture conditions

**Figure 1:**
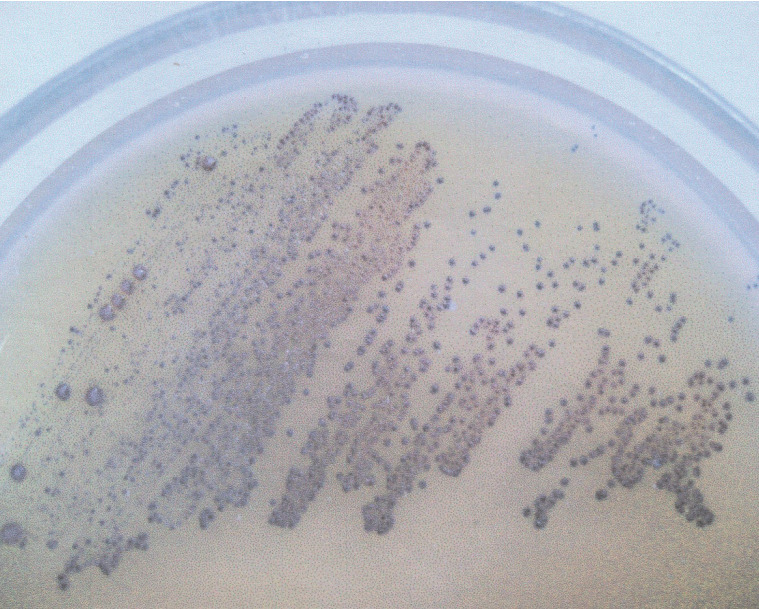
H_2_O_2_-producing *Lactobacillus acidophilus* strain (note the blue color of grown-up colonies on TMB-Plus agar in a 48 h aerobic culture with supplementation of 5% CO_2_, at 37 °C.

The following bacterial strains were used: *Streptococcus mutans* (ATCC 25175) and 2 strains of *Lactobacillus acidophilus* - MT (H_2_O_2_-producing) and VF (H_2_O_2_-not producing). *S. mutans* was cultured on Schaedler agar (Sigma, MO, USA) with 5% red sheep cells and incubated for 48 hours at 37 °C in anaerobic conditions. *L. acidophilus* strains were isolated in Biochemistry Research Laboratory Ohio University, Athens, USA from unstimulated whole saliva of healthy adults and cultured on Rogosa agar (Sigma, MO, USA) at 37 °C for 48 hours in anaerobic conditions. Selected strains were identified using API CHL (bioMerieux, SA, France). The capacity of hydrogen peroxide production among *Lactobacillus* strains was defined in culture of the obtained isolates in the presence of 5% CO_2_ at the temperature of 37 °C for 48 hours in a differentiating medium, TMB Plus agar (Sigma, MO, USA), according to Rabe and Hillier 13. At the same time, H_2_O_2_-producing *L. acidophilus* strain (ATCC 4356) constituted positive control for the tests. Development of an altered colour of the growing colonies (appearance of a blue colour) indicated the production of hydrogen peroxide (Figure 1).

### EPS production and isolation

Bacteria *S. mutans* and *L. acidophilus* isolates were inoculated in tryptic soy broth (TSB; Sigma, MO, USA) and incubated at 37 °C for 48 h in anaerobic conditions. Subsequently the cultures were centrifuged at 8000 × g at 4 °C for 10 min. The supernatants of bacterial cells were collected. Precipitation of EPS from each supernatant was provided using cold 96% ethanol (-20 °C, 1:2 v/v) stored overnight at 4 °C. Samples were centrifuged at 4 °C, 8000 × g for 10 min. Precipitates were collected, dissolved in distilled water, mixed and re-precipitated under the same conditions 14, 15. Subsequently EPS samples were centrifuged at 4 °C, 8000 × g for 10 min, mixed with PBS, digested with proteinase K (37 °C for 1 h) and dialysed against distilled water at 4 °C for 48 h. The EPS samples were stored at -20 °C. Total carbohydrates number was measured colourimetrically by phenol-sulfuric acid method in 96-well microplates 16. Absorbance level (A) was read at 490 nm using microplate reader.

### Cell cultures

Human gingival fibroblasts (HGF-1, ATCC CRL-2014) were cultured in Dulbecco's Modified Eagle Medium (DMEM; Gibco, NY, USA) supplemented with 10% heat-inactivated foetal bovine serum (FBS; Gibco, NY, USA) and with antibiotics (penicillin 100 U/ml and streptomycin 20 mg/ml; Sigma) at 37 °C in humidified 5% CO_2_ incubator. The media were replaced three times a week. Two hours before the experiments of testing ATP levels in fibroblasts, cell monolayers were washed and fresh medium was added.

### ATP assay

ATP content of gingival fibroblasts was evaluated using a luminescence test (CellTiter-Glo Luminescent Cell Viability Assay; Promega). The studies in three repetitions, were conducted in culture medium in the presence of a buffered physiological saline (50 µl PBS/0.5×10^6^ HGF-1 cells/450 µl culture medium) - the control, and in the presence of each isolated EPS (50 µl EPS/0.5×10^6^ HGF-1 cells/450µl). In preliminary experiments it was confirmed that EPS in concentration of 1 mg/ml DMEM was a maximum dose for ATP levels alterations in gingival fibroblasts. The prepared cells were incubated for 24 hours in humidified 5% CO_2_ incubator at 37 °C. Then, the cells were rinsed with the buffered physiological saline (PBS) and tested for ATP according to the Promega protocol (http.//:promega.com). The results were read using a luminometer (GloMax, Promega). The light emitted in the presence of ATP was quantitated in relative light units (RLU). The intensity of emitted light quants was directly related to ATP content in the tested sample.

### Viability of gingival fibroblasts

The viability testing in HGF-1 gingival fibroblasts took advantage of the Live/Dead Viability/Cytotoxicity Kit (Invitrogen, USA) fluorescence test. The studies were conducted in Lab-Tek Chamber Slide (Nunc) culture chambers in PBS - control (50 µl PBS/0.5×10^6^HGF-1 cells/450 µl culture medium), or in the presence of each EPS obtained from cultures of selected bacterial strains (50 µl EPS/0.5×10^6^ HGF-1 cells/450 µl culture medium). The medium involved DMEM (Gibco, NY, USA) solution, enriched with 10% foetal bovine serum (FBS, Gibco, NY, USA). The prepared cells were incubated for 24 hours in humidified 5% CO_2_ incubator at the temperature of 37 °C. Subsequently, the cells were washed in the culture medium and tested for their viability. The readouts were made at zero time and following 6, 12, 18 and 24 hours, using a Nikon Eclipse E200 fluorescence microscope (magnification of 1000×).

### Statistical analysis

Results obtained in the tests were analysed using the computer software PQStat 1.8.0. Data distribution normality was controlled with Shapiro-Wilk test. For the comparative analysis of ATP levels alterations in time ANOVA test was applied for repeated measures. The comparison of respective results between the tested groups was made on the basis of contrasts analysis for One-Way ANOVA. In every test, hypotheses were verified at the significance level of p=0.05. Data are presented as the mean ± standard deviation (sd).

## Results

### ATP levels in gingival fibroblasts (HGF-1)

**Figure 2:**
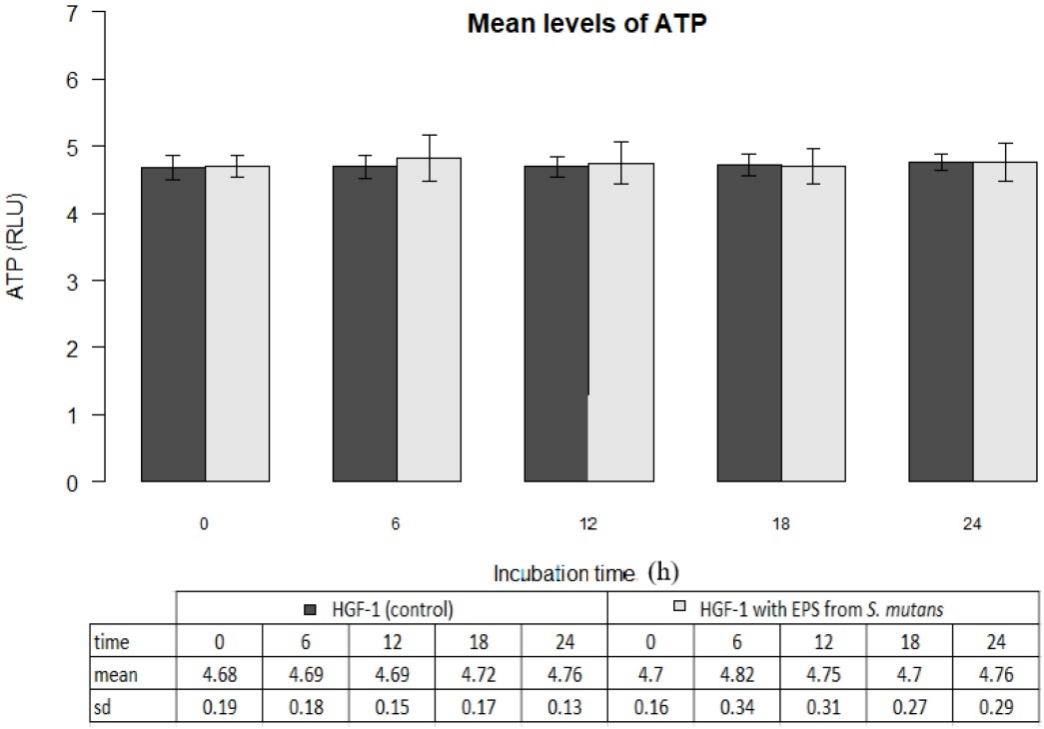
Mean levels of ATP (luminescence in millions of RLU) in cultures of gingival fibroblasts (HGF-1) at zero time and following 6, 12, 18 or 24 h incubation with EPS from *S.mutans.*

The mean ATP level in gingival fibroblast cultures (control) at zero time amounted to 4.68±0.19 million RLU. The result was not statistically different from the mean ATP values determined within the next 6, 12, 18 or 24 hours (p>0.05). The results are shown in Figure 2.

### EPSs of Streptococcus mutans activity

EPSs of *Streptococcus mutans* (ATCC 25175) did not change ATP levels in gingival fibroblasts (p>0.05) The mean ATP level in the presence of EPSs after 6 hours of incubation was 4.82 ± 0.34 and within 24 hours corresponded to the control values (Figure 2).

### EPS_s_ of oral lactobacilli activity

**Figure 3:**
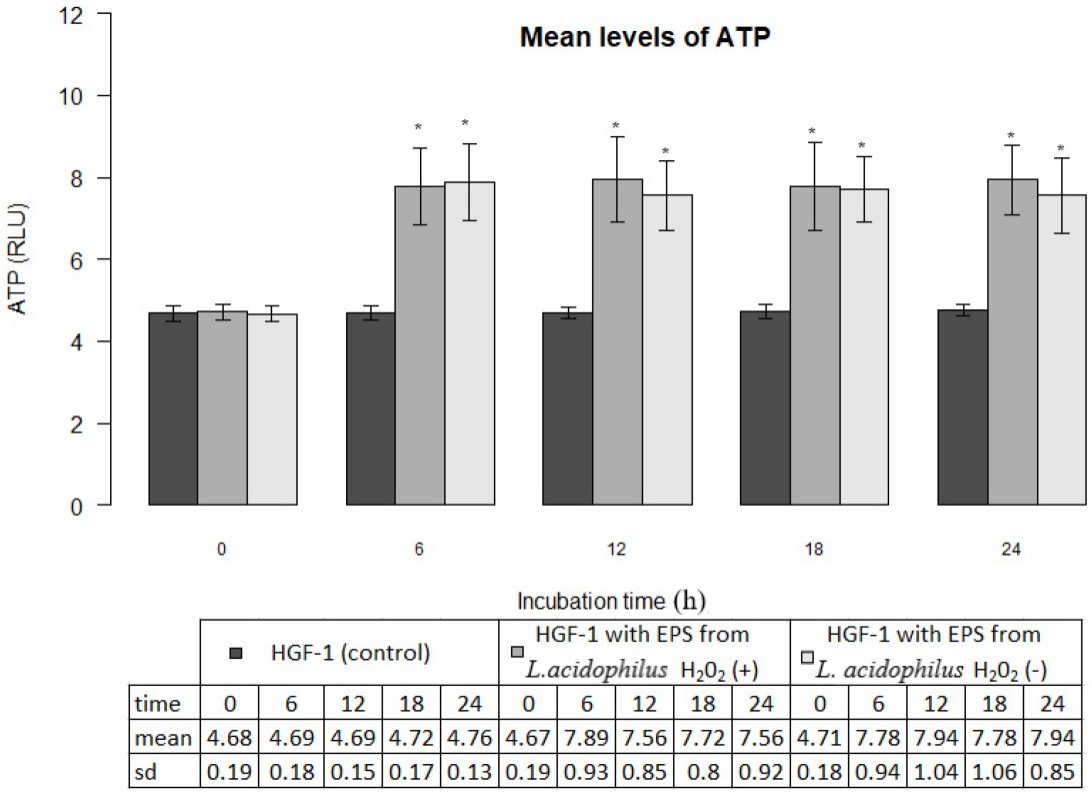
Mean levels of ATP (luminescence in millions of RLU) in cultures of gingival fibroblasts (HGF-1) at zero time and following 6, 12, 18 or 24 h incubation with EPS from *L. acidophilus* H_2_O_2_ (+) or *L.acidophilus* H_2_O_2_ (-); * marked significant difference compared to zero time.

EPSs produced by *L. acidophilus* strains significantly increased ATP levels in gingival fibroblasts as compared with control cultures (p<0.001). The mean ATP levels in fibroblasts cultures in the presence of EPSs of H_2_O_2_-producing *L. acidophilus* and H_2_O_2_-not producing *L. acidophilus* already after 6 hours amounted to 7.89±0.93 million RLU and 7.78±0.94 million RLU respectively, whereas those values, at all times of the test were not statistically different (p>0.05). The induced effect of EPSs persisted for a period of 24 h was presented in Figure 3.

### Viability of gingival fibroblasts

**Figure 4:**
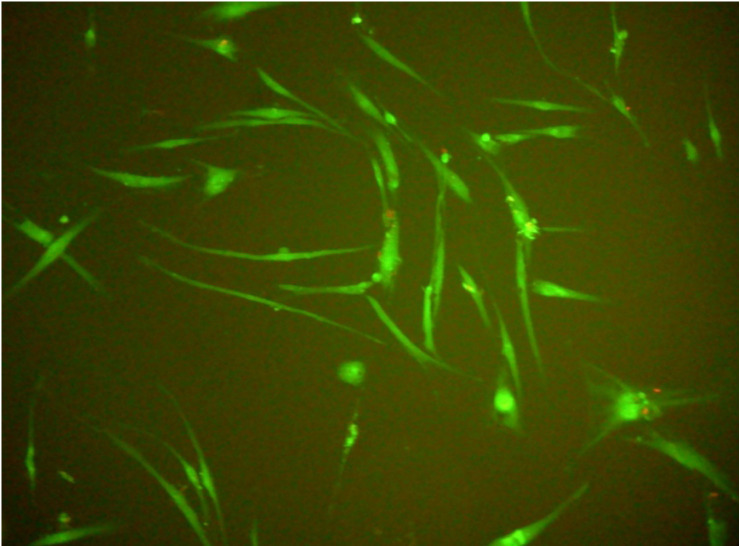
Human gingival fibroblasts (HGF-1)-fluorescence test (live/dead; viability/cytotoxicity kit).

**Figure 5:**
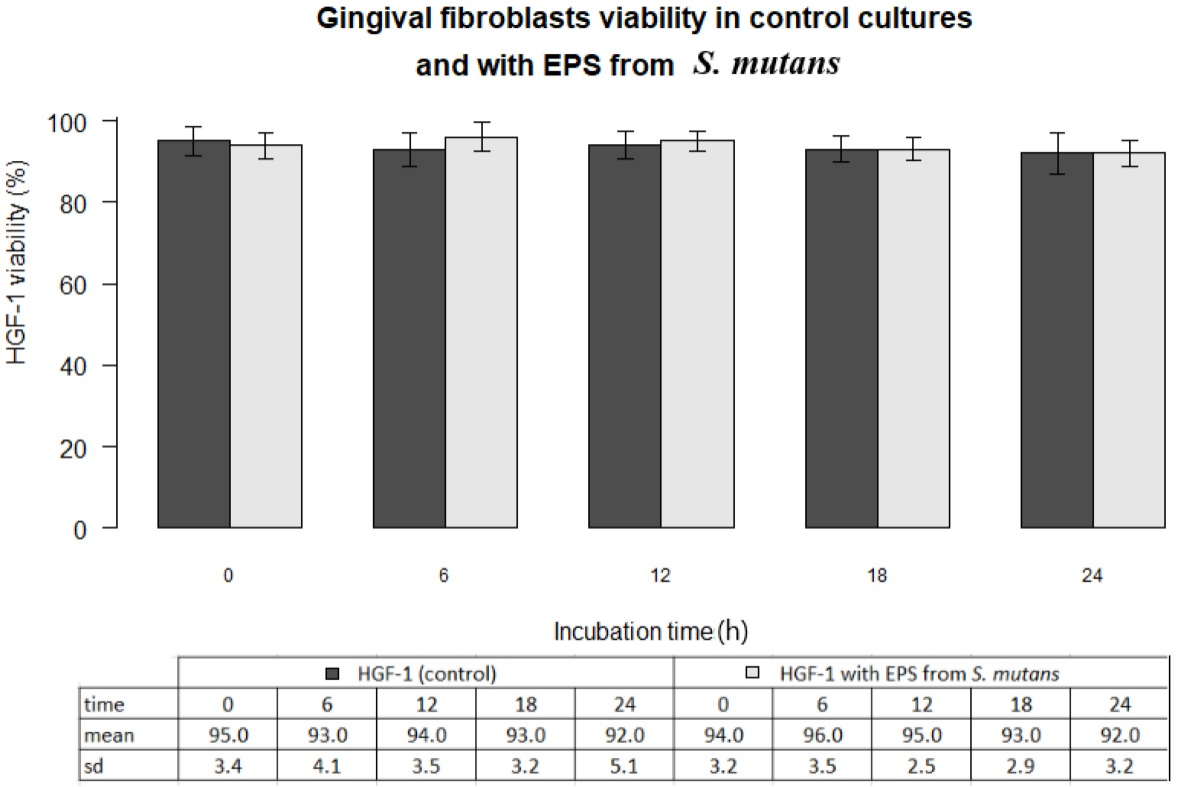
Gingival fibroblasts (HGF-1) viability (%) in control cultures and with EPS from *S. mutans* at zero time and following 6, 12, 18, 24 h incubation.

**Figure 6:**
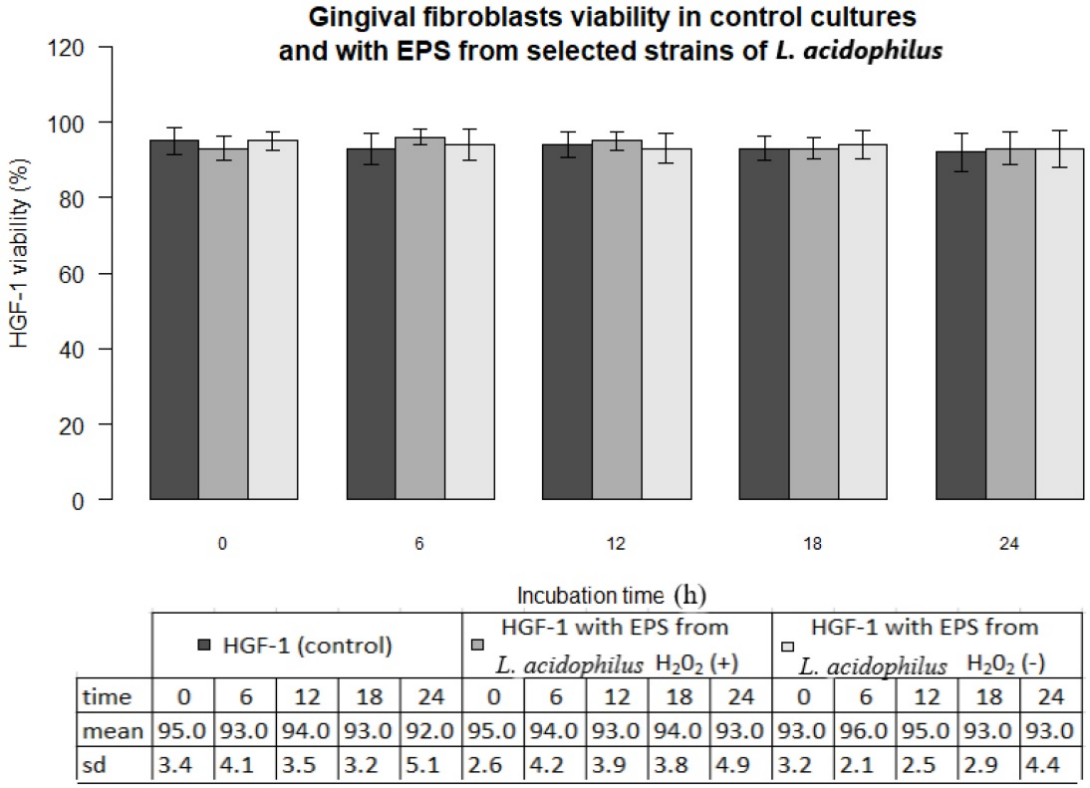
Gingival fibroblasts (HGF-1) viability (%) in control cultures and with EPS from from *L. acidophilus* H_2_O_2_ (+) or *L.acidophilus* H_2_O_2_ (-) at zero time and following 6, 12, 18, 24 h incubation.

In all experiments 92-96% gingival fibroblasts were viable cells, indicating green fluorescence with the use of Live/Dead Viability/Cytotoxicity Kit (Figure 4). No significant differences were observed between the viability of fibroblasts in control cultures and in the presence of EPSs from *S. mutans* or selected oral lactobacilli (p>0.05)*.* The results obtained at zero time and in next 6, 12, 18 or 24 hours are shown in Figure 5 and 6.

## Discussion

This work analyses the effect of EPSs from cariogenic bacteria - *S. mutans* and *L. acidophilus* on ATP levels in human gingival fibroblasts. EPSs produced by the bacteria allow the formation of dental plaque (cariogenic biofilm) and are recognized as an essential virulence factor associated with dental caries 17, 18, 19. At the same time it is noted that dental plaque accumulation located at and below the gingival margins induces gingival inflammation 20, 21. This process starts chronic periodontitis which, in the consequence, leads to the destruction of periodontium 9, 22. The studies presented in this paper were conducted with the use of human gingival fibroblasts which constitute the dominant cells of the periodontium connective tissue and ensure integrity of its structure 23, 24. The cellular adenosine triphosphate (ATP) level represents a significant exponent of cell metabolic activity and viability 25. *In vitro* tests indicated that proliferative activity of fibroblasts is induced by the extracellular ATP 26. A decrease in cellular ATP production provides metabolic conditions leading to cell death by different mechanisms, including apoptosis, autophagy, or necrosis 27, 28. Several techniques are used to estimate ATP concentration in cells 29, 30. However, the bioluminescence method provides the most sensitive and specific measurement 31, 32. It has already been well documented that the emitted light intensity is linearly related to the ATP concentration 33. These data justify the use of the above method in this work. The research did not prove the effect of EPS from *S. mutans* on ATP production in gingival fibroblasts. EPS secreted by *S*. *mutans* constitute monosaccharides, mainly α-D-glucans, which play a key role in the formation of dental plaque, mediating in cariogenic effect 34. It is already known that oral streptococci, including *S. mutans*, may colonize both dental enamel and oral cells. There was also proven the importance of *S. mutans* biofilm in promoting these bacteria for adherence and invasion of human gingival cells 35. However, there is no data presenting direct toxic effect of secreted EPS (α-D-glucan) from *S. mutans* on cells, which was not demonstrated in this work, either. Our results show that EPS (α-D-glucan) *S. mutans* does not affect the metabolic activity of gingival fibroblasts. Cytotoxicity of EPSs both from *S. mutans*, and from *L. acidophilus* towards fibroblasts was not demonstrated, either. Their viability in all cultures was not significantly different. On the other hand the relationship between dental plaque and destruction of periodontal tissues has already been well-documented 21, 22. The earliest pathological changes associated with the development of dental plaque and its location at the gingival margin existing in gingival tissue may be the result of direct microbial action through their secreted products, or indirect action, in the result of bacterial induction of inflammatory response 36. However, considering the data presented in this work, a conclusion can be drawn that α-D-glucan is not a direct factor destructing gingival tissues, so *per se* it does not play a role in initiating periodontal disease.

Whereas, EPSs from *L. acidophilus* isolates, irrespective of their ability to secrete H_2_O_2_, significantly increased ATP levels in gingival fibroblasts, which is demonstrated in this work for the first time. This effect may be caused by β-D-glucans which, as it has been documented, are the constituents of EPSs produced by some strains of *L. acidophilus* 37, 38. At the same time, β-D-glucans are considered as biological response modifiers 38, 39, 40. Biological effects of β-D-glucans are influenced by their degree of branching, chain length, and tertiary structured 41. It has been well-documented that EPS derived from *L. rhamnosus* KL37 demonstrates anti-inflammatory potential. The recent research show, that the above EPS may control T lymphocytes activity in various inflammatory diseases 42. It was also demonstrated that immunoregulatory properties of EPSs from *Lactobacillus* are strongly strain specific 43, 44. The results obtained in this work are consistent with our prior observations 12 and correspond to other data indicating that EPSs from some lactobacilli may protect human cells 38, 40. In the context of the above, it may by suggested that by promoting ATP synthesis in gingival fibroblasts EPSs from selected *L. acidophilus* strains stimulate their activity and process repairing. So, it seems likely that EPSs from selected strains of oral lactobacilli may limit the development of periodontitis.

At the same time, the diversity of *L. acidophilus* strains determined by the ability to secrete H_2_O_2_, which may be of particular significance in the pathogenesis of dental caries and periodontitis, is well-known 45, 46. In this work, the effect on EPSs fibroblasts deriving from two different strains of *L. acidophilus* (H_2_O_2_-producing and H_2_O_2_-not producing) was not different, and did not confirm the strain specific activity of EPS. In the context of the above data it may be assumed that EPSs of both strains of one species have the same structure determining the same properties. So the tests prove that bacterial EPS is mainly species specific. However, strain specificity of EPS is also possible, which is determined by the synthesis of its altered structure among some strains of the same species.

In conclusion, the data obtained in this work demonstrated that EPS from *S. mutans* does not cause changes in ATP levels in HGF-1 and does not affect their viability, therefore it cannot constitute *per se* a pathogenic factor in initiating gingivitis associated with dental plaque. Whereas, EPSs deriving from some strains of oral lactobacilli cause an increase of ATP production in fibroblasts and therefore stimulate their activity which may demonstrate protective effect towards those cells.
